# Iranian temporal changes in semen quality during the past 22 years: A report from an infertility center

**DOI:** 10.18502/ijrm.v18i12.8027

**Published:** 2020-12-21

**Authors:** Serajeddin Vahidi, Mohammad Reza Moein, Fatemeh Yazdinejad, Saeed Ghasemi-Esmailabad, Nima Narimani

**Affiliations:** ^1^Research and Clinical Center for Infertility, Yazd Reproductive Sciences Institute, Shahid Sadoughi University of Medical Sciences, Yazd, Iran.; ^2^Abortion Research Center, Yazd Reproductive Sciences Institute, Shahid Sadoughi University of Medical Sciences, Yazd, Iran.; ^3^Hasheminejad Kidney Center, Iran University of Medical Science, Tehran, Iran.

**Keywords:** Infertility, Semen quality, Temporal changes.

## Abstract

**Background:**

Despite numerous reports about temporal changes in semen quality from all over the world, the debates continue. The latest systemic review has shown an overtime decrease in semen quality worldwide.

**Objective:**

To assess the temporal changes in the semen quality among Iranian population referred to an infertility center.

**Materials and Methods:**

In this retrospective cross-sectional study, semen parameters including concentration, motility, and morphology were compared between Iranian men reffered to Research and Clinical Center for Infertility, Yazd between 1990 to 1992 (group 1, n = 707) and 2010 to 2012 (group 2, n = 1108). Demographic characteristics and semen analysis were collected from the records. The effect of age on semen parameters was also investigated.

**Results:**

Despite the increase in sperm concentration l in group 2, sperm with normal morphology decreased significantly (p < 0.001). Grade-A motility decreased (p < 0.001), grade B motility increased (p < 0.001), and grade C and D motile sperm remained constant (p = 0.303 and p = 0.315, respectively). Also, no significant correlation between the age and semen parameters were observed.

**Conclusion:**

This study showed inconsistent temporal changes in the participant semen quality. Significant temporal decline were obtained between various semen parameters, sperm morphology and grade A motility. These results should be further evaluated by larger studies in the future.

## 1. Introduction

More than 15% of couples are estimated to be suffering from infertility and its burdens (1). Besides its negative role in couple relationship and quality of life, it may impose a significant economic burden on the affected family (2). In 2000, more than 18 billion dollars were expended for assisted reproductive technique in the United States (3). Beside, male factor plays an important role in more than 50% of infertility cases; an evaluation of male fertility potential through semen analysis is the cornerstone in the andrology laboratories.

A meta-analysis by Carlsen and colleagues denoting significant decline in sperm count from 1938 to 1991 has attracted much media and scientific attention (4). Since their meta-analysis included heterogenous studies, the accuracy of their findings has been questioned by others (5, 6). Nevertheless, till date, the Carlson study continue to be the source of many debates. Several studies from different countries have tried to detect trends in their geographic region and this has led to contradictory results. Geoffroy-Siraudin and colleagues and shine and co-workers in the retrospective studies on 10,932 French and 975 New Zealander participants, respectively, showed a significant decrease in semen quality over time (7, 8). The decrease in semen quality is likely to be due to environmental pollution (due to industrialization) or urban life style (smoking, obesity, mobile phone, etc.). However, in contrary, Fisch and colleagues and Berling and coworkers in the retrospective studies on 1,283 American and 718 Swedish semen samples, respectively, did not report a temporal deterioration in the semen quality (9, 10).

The discrepancy between these studies may be due to differences in study design, geographic regions, ethnicity, methods for statistical analysis, and selection bias (6). For example, in term of selection bias, participants from an infertility center, sperm donor bank, or military service are not the true representative of the general population. According to Fisch and co-workers, semen sample of infertile couples due to female factors might have the closest similarity to general population (11).

These contradictory results prompt us to assess the Iranian temporal trends in the semen quality of men who were referred to the Yazd infertility center due to female factors. This study can be a baseline for future larger studies from Iranian community.

## 2. Materials and Methods

In this retrospective cross-sectional study, the semen quality of men who were referred to the research and clinical center for infertility, Yazd, Iran, was compared between 1990 and 1992 (group 1, n = 707) and 2010 to 2012 (group 2, n = 1108). In order to decrease the selection bias, only the male partners of infertile couples with known female factors (tubal obstruction, endometriosis, etc.) were chosen. The patients with varicocele or cryptorchidism, known history of hormonal derangements and high-risk sexual behavior (if recorded) were excluded from our survey.

### Semen collection 

Participants' sperm samples were taken by masturbating in the laboratory or at their preferred location 2 to 7 days after the last intercourse. All samples were analyzed by conventional manual methods in 30-60 min of ejaculation according to the 1987 guidelines of world health organization (12) for the first group and 1999 of WHO (13) for the second group. Next, sperm motility was evaluated using a phase-contrast microscope (Olympus, Tokyo, Japan) and expressed as quick (grade A), slow (grade B), non-progressive (grade C), and immotile (grade D). The sperm morphology was then assessed with light microscope (Olympus, Tokyo, Japan) after Diff-Quick stain on air-dried smears.

### Ethical consideration

The study was approved by the ethical committee of research and clinical center for infertility, Shahid Sadoughi University of Medical Sciences, Yazd, Iran (IR.SSU.RSI.REC.1397.030).

### Statistical analysis

Data were analyzed using the SPSS version 17 software (SPSS Inc, Chicago, Illinois, USA) and the results were presented as mean ± standard deviation (SD). A paired *t* test was used for the data analysis and p < 0.05 was considered as the level of significance.

## 3. Results

Finally, the semen quality of 1815 men referred to the clinic were analyzed and compared between groups. Table I present the patients' characteristics and variables. Results showed that mean percentage of spermatozoa in grade A and B motility significantly decreased and increased respectively, while grades C and D motility remained constant. Sperm morphology had the most impressive result, in which the percentage of spermatozoa with normal morphology decreased significantly.

Since the mean age difference between the two groups was statistically significant, the effect of this factor on semen quality was evaluated using the Pearson's correlation test (Table II).

**Table 1:** Comparison of temporal changes in semen quality between the studied groups


**Variable** **Year**	**1990-1992**	**2010-2012**	**P-value**
**No. of patients**	707	1108	
**Age (Yr)**	33.00	34.00	0.001
**Volume (ml)**	2.94 ± 1.09	2.83 ± 1.11	0.052
**Concentration (Million/ml)**	84.48 ± 1.6	95.55 ± 45.7	0.000
**Grade A (%)**	38.67 ± 16.18	30.59 ± 9.79	0.000
**Grade B (%)**	21.34 ± 10.2	30.30 ± 7.8	0.000
**Grade C (%)**	9.27 ± 5	9.01 ± 5.23	0.303
**Grade D (%)**	30.71 ± 12.2	30.18 ± 10.15	0.315
**Normal morphology (%)**	62.2 ± 12.3	44.44 ± 16.31	0.000
Data presented as Mean ± SD. Paired *t* test			

**Table 2:** Correlation between semen parameters and age in the two groups


**Parameters**	**Year 1990-1992**		**Year 2010-2012**	
	**Correlation with age**	**P-value**	**Correlation with age**	**P-value**
**Concentration**	0.032	0.39	0.048	0.11
**Volume**	-0.067	0.75	0.028	0.35
**Rapid motility (Grade A)**	-0.075	0.046	0.049	0.1
**Slow motility (Grade B)**	0.024	0.51	-0.047	0.12
**Non progressive (Grade C)**	0.017	0.65	-0.063	0.037
**Immotile (Grade D)**	0.073	0.052	0.034	0.26
**Normal morphology**	-0.067	0.07	0.040	0.18
Paired *t* test				

## 4. Discussion

In this study, we evaluated the time-dependent changes in semen quality of Iranian men, referred to an infertility center due to female factors. This group of participants might have the closest similarity to the general populations (11). Nevertheless, our result should be cautiously attributed to the general populations. As mentioned in the result, while there was an increase in the sperm count, the percentage of morphologically normal and rapid motile sperm decreased significantly. It has been demonstrated that the decline in sperm morphology (14) and motility (15) is associated with longer time to pregnancy and subsequent subfertility or even infertility.

There was also a statistically significant difference between the mean ages of the two groups. Therefore, using the Pearson's correlation test, we have further evaluated the effect of age on semen quality. As shown in Table II, there was no significant correlation between the age and the semen parameters. Although the two parameters including quick and non-progressive motility were statically correlated with age, these correlations seemed not to be important in the clinical practice. It might be due to the large sample volume that yields in statistically and not clinically important correlation. Our results are in line with those reported by Mukhopadhyay and co-workers and Selevan and colleagues who showed no change or even increased sperm count and decreased sperm motility in groups of Indian and Czech male population (5, 16, 17), respectively. Also, Jorgensen and colleagues in a prospective study of 4,867 Danish men (general population) showed that although sperm concentration significantly increased over a period of 15 yr, only 23% of the included participants had optimal seminal value (18).

Further, in a retrospective study on 26,609 French male participants by Rolland and co-workers, between 1989 and 2005, the sperm concentration and morphology showed a significant decline over time (19). In similar line, Geoffroy-Siraudin and coworkers in a retrospective study on 10,932 patients from Marseille, France stated that all semen parameters (including sperm concentration, motility, and morphology) decreased over a period of 20 yr (7). Higher level of environmental pollutant in their region (Marseille is an industrialized city with probable high level of environmental pollution) may explain the differences between their and our results. However, in contrary, Itoh and co-workers and Costello and colleagues in a retrospective study on 457 Japanese men (healthy volunteer) and 448 Australian participants (for sperm Donation), respectively, reported no deterioration in the sperm concentration (20, 21). The type of sample selection (healthy volunteer), smaller sample size (in comparison with ours), and regional differences may have contributed to such differences.

Therefor, the discrepancy between opponent and proponent may originate from selection bias (samples from infertility center, sperm donor bank, military service, etc.), differences in geographic region, paternal age, ethnicity, abstinence time, sample size, and analytic methods. To the best of our knowledge, despite the previous reports about Iranian infertility rate (22), the present study is by far the first report on Iranian semen quality trends over time (from an infertility center). The etiologies of these changes remain to be elucidated. The rapid evolution of our economy from traditional agriculture to modern industries and life style changes, environmental pollution, obesity, tobacco, and drug abuse might play a significant role in the over-time changes observed in semen quality in our geographic region. We are aware of possible selection bias in our study. Considering the great percentage of referral from all over the country, the effect of geographic region as a confounder should also be considered. The long duration of our study, changes of laboratory technician or their increased experience in this period of time may all cause Inter- or intra- individual bias. We think that our results should be further evaluated with future prospective studies.

## 5. Conclusion

In this study, we have mentioned a temporary decrease in some semen parameters like sperm motility and morphology despite increase in sperm concentration from an Iranian infertility center. The reason for these changes remain to be elucidated with further studies.

##  Conflict of Interest

No conflict of interest
